# Stakeholder perspectives on the Emirati board comprehensive clinical examination: a mixed-methods multi-stakeholder evaluation

**DOI:** 10.3389/fmed.2026.1900166

**Published:** 2026-10-14

**Authors:** Asma Fatima Syeda, Ibrahim Elhassan, Fouzia Shersad, Hadeel Aboueisha, Abdul Muqsit, Mohammed Al-Houqani, Azhar T. Rahma, Mohi Eldin Magzoub

**Affiliations:** 1 National Institute for Health Specialties, Al Ain, United Arab Emirates; 2 Internal Medicine Department, College of Medicine and Health Sciences, United Arab Emirates University, Al Ain, United Arab Emirates; 3 Institute of Public Health, College of Medicine and Health Sciences, United Arab Emirates University, Al Ain, United Arab Emirates; 4 Department of Medical Education, College of Medicine and Health Sciences, United Arab Emirates University, Al Ain, United Arab Emirates

**Keywords:** high-stakes clinical assessment, objective structured clinical examination, stakeholder perceptions, mixed-methods research, postgraduate medical education, quality improvement

## Abstract

**Background:**

High-stakes clinical examinations play a critical role in postgraduate medical training by assessing readiness for independent specialist practice. While hybrid Objective Structured Clinical Examination (OSCE)-style assessments are increasingly used to evaluate integrated clinical competence, evidence incorporating the perspectives of multiple stakeholders remains limited, particularly in large-scale specialist certification examinations. Multi-stakeholder evaluations may provide valuable insights into examination quality, implementation, and stakeholder experience.

**Objective:**

To evaluate stakeholder perceptions of the Emirati Board Comprehensive Clinical Examination (CCE) across key domains, including orientation, logistics, educational value, task clarity, examiner behaviour, and overall examination experience, and to identify implementation factors that may influence perceived examination quality.

**Methods:**

A convergent mixed-methods study was conducted using repeated cross-sectional stakeholder feedback collected across multiple CCE examination sittings between November 2024 and January 2026. Quantitative data were obtained from post-examination surveys completed by examinees (*n* = 368), examiners (*n* = 390), and quality assessors (QA; *n* = 88), supplemented by post-examination debriefing notes from all stakeholders. Quantitative data were analysed using descriptive statistics and group comparisons, while qualitative data underwent thematic analysis. Findings from both data strands were integrated during interpretation.

**Results:**

Examiners and quality assessors reported consistently positive perceptions of the examination (mean scores 4.4–4.8 out of 5), whereas examinees reported more moderate ratings (overall mean 3.88 out of 5), particularly regarding orientation, logistics, and task clarity. Qualitative findings complemented these results by identifying perceived variability in task instructions, time allocation, examiner interactions, and simulated patient performance. Integration of quantitative and qualitative findings indicated broad agreement regarding the educational value and overall organization of the examination, while also highlighting differences between system-level evaluations and examinees’ lived experiences.

**Conclusion:**

The Emirati Board CCE was generally perceived as a well-organized and educationally valuable assessment by all stakeholder groups. However, examinees reported greater variability in their examination experience than examiners and quality assessors, particularly in domains related to orientation, logistics, and task clarity. These findings suggest that enhancing consistency in examination implementation may improve stakeholder experience, perceived fairness, and future quality improvement initiatives in high-stakes clinical assessment.

## 1 Introduction

High-stakes clinical examinations are fundamental components of postgraduate medical education, providing evidence that trainees have achieved the competencies required for independent specialist practice. Objective Structured Clinical Examinations (OSCEs) have become one of the most widely adopted approaches for assessing clinical competence because they enable standardized evaluation of multiple domains, including clinical reasoning, communication, professionalism, and clinical decision-making. Careful examination design, blueprinting, standardization, and quality assurance are essential to support fair and defensible assessment processes in high-stakes settings (1–5).

Contemporary postgraduate assessment systems have evolved beyond traditional OSCE formats toward hybrid clinical examinations that combine structured oral discussions, simulated patient encounters, data interpretation, and scenario-based clinical reasoning. These integrated assessment models aim to better reflect authentic clinical practice and assess higher-order reasoning and decision-making rather than isolated clinical tasks alone (4, 6, 7).

Although psychometric evidence remains central to evaluating assessment systems, stakeholder experiences also provide valuable information for quality assurance and continuous improvement. Examinees, examiners, and quality assurance personnel each observe different aspects of examination implementation, including orientation, task clarity, logistics, examiner behaviour, time allocation, and simulated patient performance. Understanding these experiences can identify operational strengths and areas for improvement that may influence stakeholders' perceptions of examination quality and fairness, even when psychometric characteristics are not directly evaluated (8–13).

The Comprehensive Clinical Examination (CCE), administered by the National Institute for Health Specialties (NIHS) in the United Arab Emirates, is a hybrid OSCE-style examination used for specialist certification across multiple medical and dental disciplines. The examination integrates structured oral discussions, simulated patient encounters, and scenario-based clinical assessments using standardized station formats designed to evaluate integrated clinical competence required for independent specialist practice (4, 5, 7).

Published evidence evaluating postgraduate licensing examinations within the Gulf region remains relatively limited. A recent study from the United Arab Emirates examined factors associated with resident performance in a national licensure OSCE and demonstrated the importance of examinee preparedness and examination experience in influencing outcomes (14). However, that study focused primarily on examinee-related factors and did not examine the examination system from multiple stakeholder perspectives. Similarly, most published studies internationally have evaluated OSCEs from individual viewpoints, focusing on psychometric performance, candidate feedback, examiner perceptions, or specific operational aspects of examination delivery, rather than integrating multiple stakeholder perspectives within a single evaluation framework (8–11, 15).

A multi-stakeholder approach offers a broader understanding of examination quality because each stakeholder group contributes unique but complementary insights. Examinees provide information about their lived assessment experience, examiners evaluate assessment delivery and educational value, while quality assessors observe examination processes across stations. Integrating these perspectives enables areas of agreement and disagreement to be identified, providing more comprehensive evidence to support quality assurance than reliance on any single stakeholder group alone.

Accordingly, this study employed a mixed-methods, multi-stakeholder design to evaluate stakeholder perceptions of the Emirati Board Comprehensive Clinical Examination across predefined domains of orientation and information, educational value, logistics and environment, task clarity, examiner behavior, Patient Performance and overall examination experience. By integrating quantitative survey findings with qualitative stakeholder feedback, the study sought to identify implementation factors perceived by stakeholders that may inform future quality improvement of high-stakes clinical assessment.

## 2 Materials and methods

### 2.1 Study design

This study employed a convergent mixed-methods design using repeated cross-sectional stakeholder feedback collected across multiple sittings of the Emirati Board Comprehensive Clinical Examination (CCE) conducted between November 2024 and January 2026. The study sought to obtain a comprehensive evaluation of examination implementation and stakeholder experience by integrating quantitative and qualitative data collected from three stakeholder groups: examinees, examiners, and quality assessors (QA).

Quantitative data comprised structured questionnaire responses collected from examinees and examiners immediately following completion of each examination sitting, together with structured quality assurance observations completed by QA during live examination delivery. Qualitative data were obtained concurrently from open-ended comment fields embedded within all stakeholder questionnaires, including QA observation forms, and from structured post-examination debriefing discussions conducted separately with examinees and examiners immediately following each examination session. These debriefing discussions, which also included participating quality assessors, were undertaken to capture participants' overall examination experiences, clarify emerging issues, and identify strengths and opportunities for improvement.

Each examination sitting represented an independent cross-sectional evaluation of stakeholder perceptions. Data collected across all examination sittings were subsequently pooled to provide an overall multi-specialty evaluation of the CCE. As the study was designed to evaluate overall examination implementation rather than changes over time, analyses were performed on the aggregated dataset, and temporal differences between examination sittings were not examined.

Consistent with a convergent mixed-methods design (16, 17), quantitative and qualitative datasets were analysed independently using appropriate analytical methods before being integrated during interpretation. Integration was achieved by comparing areas of convergence, complementarity, and divergence between quantitative findings and qualitative themes using joint displays and narrative synthesis [22,25]. This approach enabled quantitative findings to be explained and contextualized by participants' reported experiences, providing a richer understanding of examination implementation and stakeholder perceptions than either dataset alone could provide. Figure 1 illustrates the overall workflow for integration of the quantitative and qualitative components.

**Figure 1:**
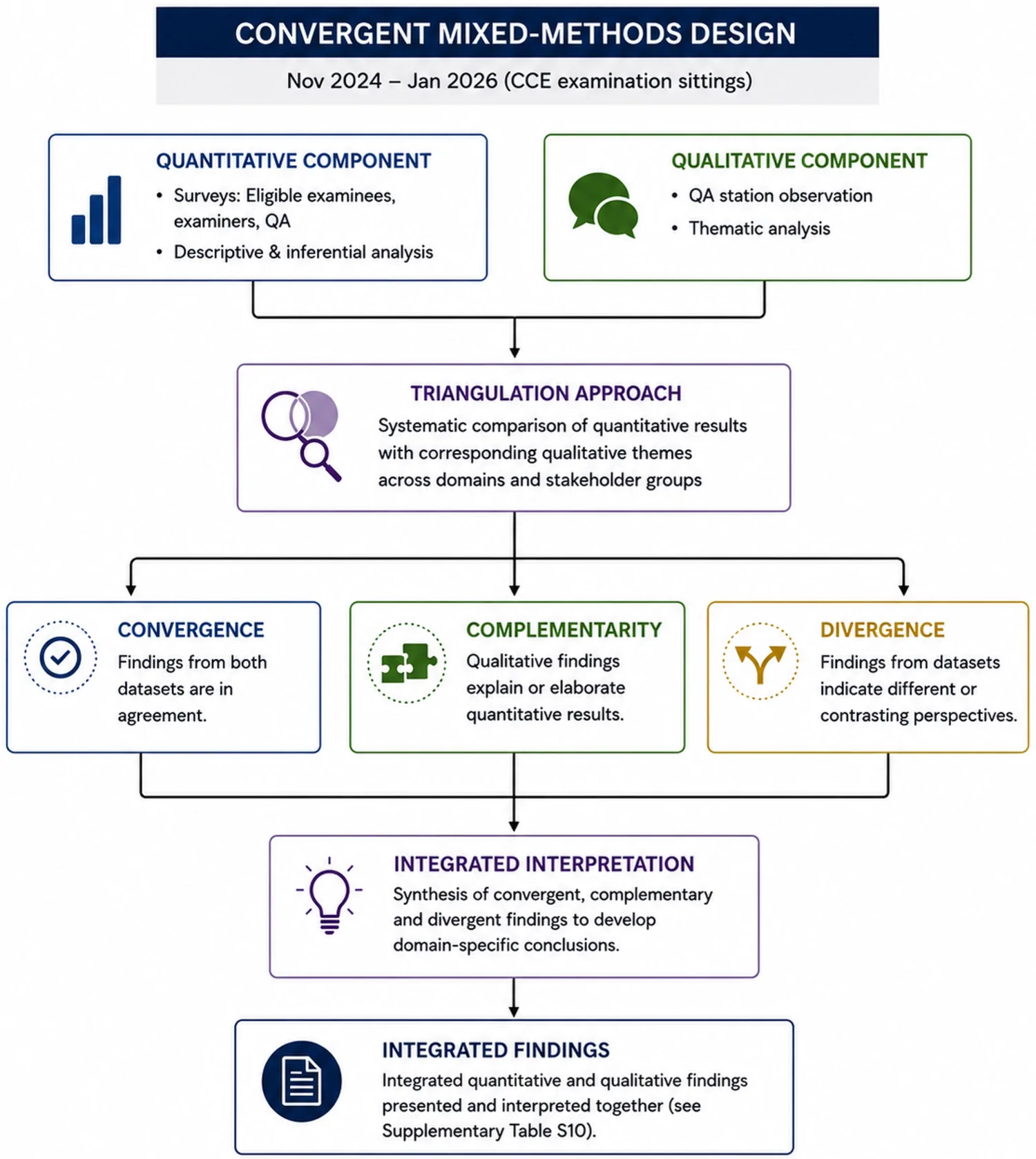
Workflow for convergent mixed-methods integration.

**Figure 2:**
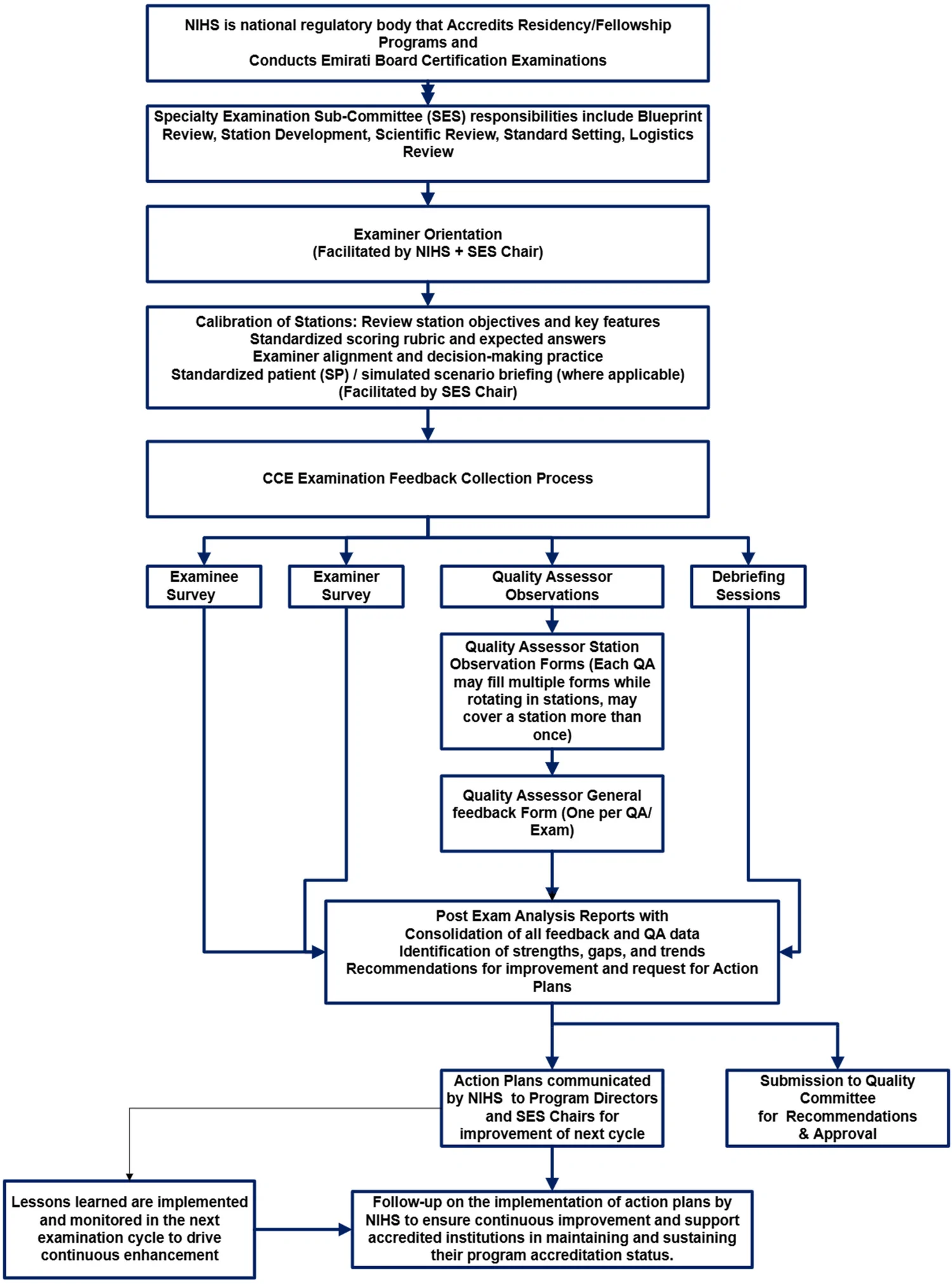
Governance and quality assurance framework of the Emirati board CCE.

**Figure 3:**
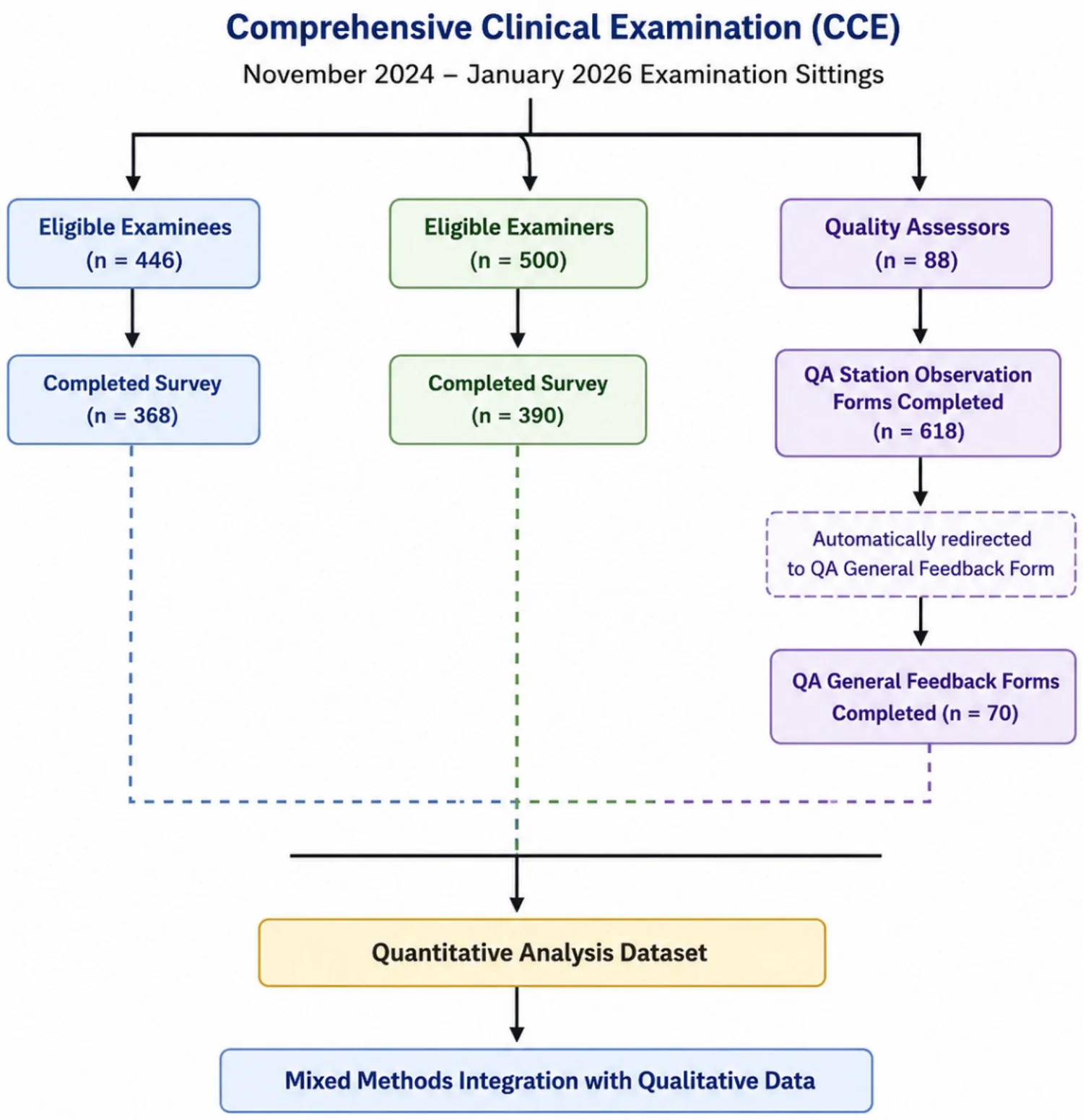
Study participant and data-source flow for the comprehensive clinical examination (CCE) evaluation.

### 2.2 Study setting, participants and sampling

The study was conducted within the National Institute for Health Specialties (NIHS), United Arab Emirates, the national regulatory body responsible for accrediting postgraduate medical and dental training institutions and programmes, and for administering the Emirati Board residency and fellowship certification examinations across all recognized medical and dental specialties in the UAE. The study included stakeholders participating in the Emirati Board Comprehensive Clinical Examination (CCE), a high-stakes hybrid OSCE-style examination that serves as the final clinical assessment for specialist board certification.

A census sampling approach was employed. All examinees, examiners, and quality assessors (QA) participating in each CCE sitting conducted between November 2024 and January 2026 were invited to complete the post-examination evaluation. The study included three stakeholder groups:

#### 2.2.1 Examinees

postgraduate medical and dental trainees enrolled in NIHS-accredited residency or fellowship programmes who undertook the CCE during the study period.

#### 2.2.2 Examiners

consultant physicians and clinical educators formally appointed by NIHS to assess candidates according to NIHS examiner eligibility criteria and requirements.

#### 2.2.3 Quality assessors (QA)

Quality assessors comprised members of the relevant Specialty Examination Sub-Committees (SES) and, where additional trained examiners were available beyond routine station requirements, senior examiners appointed by NIHS to undertake quality assessor role. The SES oversees the scientific quality of the examination, including blueprint development, station design, examiner orientation, calibration, and post-examination review. During examination delivery, QA were responsible for independently monitoring examination implementation, standardization, and adherence to established examination procedures.

Post-examination evaluations were administered using the Research Electronic Data Capture (REDCap Software) platform (18, 19). Immediately following completion of each examination, examinees and examiners were provided with a QR code linking directly to the evaluation questionnaire and were also sent an individualized survey invitation via email. Participants who had already completed the questionnaire using the QR code were instructed not to complete it again through the emailed invitation. Quality assessors completed their evaluation forms electronically during live examination delivery as part of the routine quality assurance process.

Completion of the post-examination evaluation formed part of NIHS's routine examination quality assurance programme. Examinees were requested to complete the evaluation before examination results were released, while examiners were requested to complete the evaluation before issuance of their examiner participation certificate. Quality assessors completed evaluation forms as part of their assigned quality assurance responsibilities during examination delivery. Before accessing the questionnaire, all participants were presented with an electronic consent statement requesting permission for their data to be used for research and quality improvement purposes. Only data from participants providing electronic consent were included in this study.

To encourage honest feedback, examinee and examiner questionnaires were completed anonymously, and no personally identifiable information was collected. Participants identified only their specialty and examination date. In contrast, QA forms included assessor identification because they formed part of the operational quality assurance process during live examination delivery. Prior to research analysis, QA identifiers were removed, and all datasets were de-identified.

All closed-ended questionnaire items were configured as mandatory within REDCap Software, whereas open-ended comment fields were optional. Questionnaire responses were reviewed before analysis to identify potential duplicate submissions; no duplicate records were identified. Qualitative data comprised comments voluntarily entered into the open-ended text boxes by all three stakeholder groups together with observations documented during structured post-examination debriefing discussions.

### 2.3 Examination governance and data collection

The Emirati Board Comprehensive Clinical Examination (CCE) is conducted within a structured governance framework coordinated by the National Institute for Health Specialties (NIHS) in collaboration with the relevant Specialty Examination Sub-Committees (SES). The governance and quality assurance framework is shown in Figure 2. Each SES is responsible for the scientific development and quality oversight of the examination within its specialty. Examination preparation typically begins approximately two months before the scheduled examination.

Development of the CCE follows a standardized multi-stage quality assurance process. Examination stations are developed by authorized SES members who meet NIHS eligibility requirements for examination development. To preserve examination confidentiality and maintain scientific consistency, station development is undertaken primarily by SES members. Each examination undergoes blueprint review, scientific review, content refinement, standard setting, logistical review, and final approval before implementation. Multiple levels of scientific review are undertaken to ensure alignment with programme learning outcomes, clinical relevance, assessment standards, and examination feasibility.

Examiners participate in structured orientation sessions conducted by NIHS and the SES approximately two to three days before the examination. Orientation includes review of examination policies, station procedures, assessment software, scoring rubrics, and operational processes. On the examination day, a second orientation and calibration session is conducted before candidate assessment begins. During calibration, examiners assigned to each station review station objectives, expected candidate responses, scoring criteria, marking consistency, and management of challenging examination scenarios. Where simulated or real patients and quality assessors are assigned to the station, they also participate in the calibration process to promote consistent understanding of examination procedures before candidate encounters commence.

Data were collected from four complementary sources. The participant and data-source flow is shown in Figure 3.

#### 2.3.1 Examinee surveys

Immediately after completing the examination, examinees completed a structured electronic questionnaire comprising mandatory closed-ended perception items and optional open-ended comment fields.

#### 2.3.2 Examiner surveys

Examiners completed a parallel structured electronic questionnaire immediately after the examination. Similar to the examinee survey, the instrument included mandatory quantitative items and optional open-ended comments.

#### 2.3.3 Quality assessor observations

During each Comprehensive Clinical Examination (CCE), one or more Quality Assessors (QA) were designated by the relevant Specialty Examination Sub-Committee (SES) Chair to undertake quality monitoring during examination delivery. Before the examination commenced, the SES Chair assigned QA to specific examination stations according to a predefined rotation schedule designed to ensure that all examination stations were observed during the examination.

Quality Assessors were encouraged to remain within each assigned station for at least two consecutive candidate encounters before rotating to the next station. Following each candidate encounter, the QA completed one QA Station Observation Form evaluating examination content, examiner performance, and, where applicable, simulated or real patient performance. Consequently, each Quality Assessor completed multiple QA Station Observation Forms during a single examination, depending on the number of stations and candidate encounters observed.

Upon submission of the final QA Station Observation Form, the REDCap Software platform automatically redirected the Quality Assessor to complete a single QA General Feedback Form, which captured an overall evaluation of examination organization, administration, facilities, logistics, and operational processes. All 88 Quality Assessors completed QA Station Observation Forms during the study period. However, because completion of the QA General Feedback Form occurred at the end of the electronic workflow, a small number of Quality Assessors exited the platform before submitting the final overall evaluation, most commonly because of time constraints associated with examination activities. Consequently, 70 QA General Feedback Forms were completed during the study period.

#### 2.3.4 Structured post-examination debriefing discussions

Immediately following completion of each examination, NIHS assessment staff and the relevant SES Chair facilitated structured debriefing discussions separately with examinees and examiners, with participating quality assessors also contributing to examiner debriefings. These discussions were conducted to capture participants' overall examination experiences, clarify emerging issues identified during the examination, and identify opportunities for improvement. Key discussion points and field notes were documented contemporaneously for qualitative analysis.

As part of the NIHS continuous quality improvement framework, findings from all stakeholder groups were synthesized into a comprehensive post-examination analysis report for each specialty examination. The report included quantitative survey findings, qualitative feedback, quality assurance observations, response rates, and recommended improvement actions. These reports were reviewed by the NIHS Quality Committee before examination results were released. Approved action plans were subsequently communicated to the relevant Specialty Examination Sub-Committees and monitored during subsequent examination cycles to support continuous enhancement of examination content, implementation, and standardization.

### 2.4 Survey development, expert review, and internal consistency

Four structured questionnaires were developed specifically for the Comprehensive Clinical Examination (CCE): an examinee questionnaire, an examiner questionnaire, a Quality Assessor (QA) General Feedback questionnaire, and a Station Observation QA questionnaire. Together, these instruments were designed to obtain complementary perspectives on examination organization, implementation, and stakeholder experience.

Questionnaire development was informed by established literature on clinical assessment and Objective Structured Clinical Examinations (OSCEs) (1, 3), together with existing NIHS examination evaluation instruments that had been routinely implemented within the Emirati Board quality assurance programme since late 2023. Questionnaire content was aligned with the operational structure of the CCE and evaluated predefined domains relevant to examination quality, including orientation and information, educational value, logistics and examination environment, task clarity, examiner behaviour, and, where applicable, simulated or real patient performance.

Initial questionnaire drafts were developed collaboratively by senior medical education experts, postgraduate assessment specialists, consultant clinicians, and clinical educators with extensive experience in high-stakes postgraduate assessment. The draft instruments subsequently underwent structured expert review by six subject matter experts comprising medical education specialists, assessment experts, consultant clinicians, clinical educators, and NIHS leadership. Reviewers evaluated questionnaire items for clarity, relevance, wording, feasibility, and alignment with the objectives and operational processes of the CCE. Based on this review, questionnaire items were iteratively refined to improve clarity, consistency, and usability before implementation (20).

Formal pilot testing was not undertaken as a separate research phase because the questionnaires formed part of the NIHS routine examination quality assurance programme. Instead, the instruments were introduced into routine examination practice from late 2023 and underwent iterative refinement across successive examination cycles in response to expert review, operational experience, and stakeholder feedback.

Following completion of data collection, the internal consistency reliability of all quantitative instruments was evaluated using Cronbach's alpha and McDonald's omega coefficients. Corrected item-rest correlations and reliability estimates following deletion of individual questionnaire items were also examined to assess the contribution of each questionnaire item to its respective scale. A summary of the internal consistency reliability analyses is provided in Supplementary Table S5. These analyses were undertaken to evaluate the internal consistency reliability of the study instruments and were not intended to establish questionnaire validity.

Although the stakeholder questionnaires evaluated several common aspects of examination quality, selected items were intentionally tailored to the roles and perspectives of each stakeholder group. Consequently, comparisons between stakeholder groups were limited to conceptually comparable domains, whereas stakeholder-specific items were analysed and interpreted descriptively.

### 2.5 Quantitative analysis

Quantitative analyses were performed using IBM SPSS Statistics Software for Windows, Version 31.0.1.0 (IBM Corp., Armonk, NY, USA). Additional psychometric analyses, including McDonald's omega, corrected item-rest correlations, and reliability estimates following deletion of individual questionnaire items, were undertaken using Jamovi (Version 2.7.37) (21) to verify the internal consistency of the study instruments.

All closed-ended questionnaire items were measured using five-point Likert response scales. For examinees and examiners, responses ranged from Strongly Disagree (1) to Strongly Agree (5), whereas QA questionnaires used response options ranging from Very Poor (1) to Excellent (5). Consistent with accepted practice in medical education research, multi-item Likert scale responses were analysed as approximately interval-level variables after aggregation into domain scores (22).

Questionnaire items were grouped into predefined domains reflecting the design of each instrument. For examinee and examiner questionnaires, domain scores were calculated as the arithmetic mean of the items comprising each domain, and an overall perception score was calculated as the mean of all questionnaire items completed by each respondent.

All quantitative questionnaire items were configured as mandatory within the REDCap Software platform. Consequently, missing quantitative data were minimal and occurred primarily where questions were conditionally displayed (e.g., simulated or real patient items) or were not applicable. Prior to analysis, datasets were screened for completeness, duplicate responses, and implausible values. No duplicate questionnaire responses were identified.

Internal consistency of the study instruments was evaluated using Cronbach's alpha and McDonald's omega coefficients. Corrected item-rest correlations and reliability estimates following deletion of individual questionnaire items were examined to evaluate the contribution of individual items to each questionnaire domain.

Descriptive statistics were calculated for all quantitative variables, including frequencies, percentages, means, and standard deviations. Differences in overall perception scores between examinees and examiners were evaluated using an independent samples t-test. Homogeneity of variances was assessed using Levene's test. Where the assumption of equal variances was violated, Welch's *t*-test was interpreted. Effect size was reported using Cohen's d. Statistical significance was defined as a two-sided *p* value of <0.05.

### 2.6 Qualitative analysis

Qualitative data were analysed using an inductive thematic analysis informed by the six-phase approach described by Braun and Clarke (23). Reporting of the qualitative component was informed by the Consolidated Criteria for Reporting Qualitative Research (COREQ) checklist (24). Data sources included open-ended comments from examinee, examiner, and quality assessor questionnaires, together with field notes documented during structured post-examination debriefing discussions.

Analysis was conducted by four members of the research team with experience in medical education and assessment. Initial coding was performed independently before codes were discussed collaboratively and organized into a shared coding framework. Themes were developed iteratively through comparison of stakeholder perspectives and refinement of coding decisions until agreement was reached within the research team.

To strengthen the credibility of the findings, qualitative data from multiple stakeholder groups and data sources were triangulated during analysis. Emerging themes were subsequently compared with quantitative findings during interpretation to identify areas of convergence, complementarity, and divergence across stakeholder groups.

Representative quotations were selected to illustrate major themes while preserving participant anonymity.

#### 2.6.1 Researcher reflexivity

Qualitative analysis was undertaken by members of the research team with expertise in medical education, postgraduate assessment, and clinical training. Several researchers were directly involved in the governance, development, delivery, or quality assurance of the Emirati Board Comprehensive Clinical Examination (CCE), providing valuable contextual understanding of the examination process while recognizing the potential for institutional confirmation bias.

To enhance the credibility and trustworthiness of the findings, qualitative coding was conducted independently before codes were compared and discussed collaboratively. Differences in coding and interpretation were resolved through iterative discussion until consensus was achieved. Themes were developed using data from multiple stakeholder groups (examinees, examiners, and Quality Assessors) and multiple qualitative sources, including questionnaire comments and structured post-examination debriefing field notes. Throughout the analytical process, the research team engaged in ongoing reflexive discussions regarding how their professional roles and prior involvement in postgraduate assessment might influence interpretation. Triangulation across stakeholder groups, qualitative data sources, quantitative findings, and operational quality assurance observations was used to ensure that interpretations remained grounded in participants' accounts rather than researchers' prior assumptions.

### 2.7 Use of artificial intelligence

A generative artificial intelligence (AI) large language model (ChatGPT, OpenAI, GPT-5.5; accessed July 2026) was used to assist with the preliminary organization of anonymized qualitative responses, including grouping similar comments and supporting the initial organization of large qualitative datasets. All data were fully anonymized before processing.

The AI tool was not used to generate themes, interpret findings, perform statistical analyses, or draw study conclusions. All coding decisions, theme development, interpretation of findings, manuscript preparation, and final scientific judgments were undertaken independently by the research team. All AI-generated outputs were critically reviewed, verified, and edited by the authors before inclusion in the study.

The use of AI was limited to supportive organizational tasks and complied with the journal's guidance regarding transparent disclosure of AI-assisted research. The prompts used to guide AI-assisted organization of qualitative data and the corresponding workflow are provided in Supplementary Document S11 to enhance transparency and reproducibility.

### 2.8 Ethical considerations

Ethical approval for this study was obtained from the United Arab Emirates University Social Sciences Research Ethics Committee (Approval No. ERSC_2025_6404).

The study analysed routinely collected examination quality assurance data generated as part of the National Institute for Health Specialties' (NIHS) examination governance processes. Before completing the evaluation questionnaires, all participants were presented with an electronic consent statement requesting permission for their de-identified data to be used for research and quality improvement purposes. Only data from participants providing electronic consent were included in the study.

Examinee and examiner questionnaires were completed anonymously. Although quality assessor forms contained assessor identifiers during operational quality assurance activities, all identifiers were removed before preparation of the research dataset. Data were stored securely and analysed in accordance with institutional confidentiality and data protection requirements.

As part of the NIHS continuous quality improvement framework, findings from stakeholder evaluations were routinely synthesized into post-examination analysis reports submitted to the NIHS Quality Committee. These reports informed specialty-specific action plans that were reviewed and monitored during subsequent examination cycles to support ongoing enhancement of examination quality, implementation, and standardization.

## 3 Quantitative results

### 3.1 Participant characteristics

A total of 368 examinees, 390 examiners, and 88 Quality Assessors (QA) participated in the study across multiple sittings of the Emirati Board Comprehensive Clinical Examination between November 2024 and January 2026. Response rates were 82.5%, 78.0%, and 100%, respectively.

Examinee and examiner responses represented 19 medical and dental residency and fellowship specialties. The largest examinee groups were Family Medicine (*n* = 91), Internal Medicine (*n* = 68), Pediatrics (*n* = 61), Emergency Medicine (*n* = 44), and Psychiatry (*n* = 33). Examiner participation followed a similar distribution, with Family Medicine (*n* = 106), Pediatrics (*n* = 56), Internal Medicine (*n* = 47), Emergency Medicine (*n* = 38), and Psychiatry (*n* = 25) contributing the largest numbers of respondents.

Quality Assessor data comprised two complementary datasets. During live examination delivery, 88 Quality Assessors completed 618 QA Station Observation Forms, representing repeated operational observations across multiple candidates encounters and examination stations. Following completion of the station observations, Quality Assessors were automatically redirected to complete one QA General Feedback Form, resulting in 70 completed QA General Feedback Forms providing overall evaluations of examination organization and operational processes.

### 3.2 Internal consistency of the study instruments

The internal consistency of all quantitative study instruments is summarized in Supplementary Table S5. Internal consistency reliability analyses performed using Jamovi (Version 2.7.37) indicated good to excellent internal consistency across all study instruments, supporting the reliability of the questionnaires used to evaluate stakeholder perceptions.

The examinee questionnaire demonstrated excellent internal consistency (Cronbach's *α* = 0.891; McDonald's *ω* = 0.897), while the examiner questionnaire also demonstrated excellent internal consistency (Cronbach's *α* = 0.943; McDonald's *ω* = 0.947). The General Quality Assessor Feedback questionnaire demonstrated good internal consistency (Cronbach's *α* = 0.881; McDonald's *ω* = 0.906).

Reliability analyses of the Quality Assessor Station Observation Form were performed separately for each evaluation domain. Educational Value demonstrated excellent internal consistency (Cronbach's *α* = 0.916; McDonald's *ω* = 0.921), Simulated Patient Performance demonstrated excellent internal consistency (Cronbach's *α* = 0.934; McDonald's *ω* = 0.940), and Examiner Performance demonstrated good internal consistency (Cronbach's *α* = 0.832; McDonald's *ω* = 0.847).

Across all instruments, corrected item-rest correlations exceeded 0.50, and reliability estimates following deletion of individual questionnaire items did not improve overall internal consistency, indicating that all questionnaire items contributed meaningfully to their respective scales and supporting retention of the complete questionnaires.

### 3.3 Stakeholder perceptions

Stakeholder perceptions of the Comprehensive Clinical Examination (CCE) are summarized in Table 1. Overall, all stakeholder groups evaluated the examination positively across the principal evaluation domains, although the magnitude and consistency of these perceptions differed between stakeholder groups.

**Table 1:** Comparison of stakeholder perceptions across the principal evaluation domains of the comprehensive clinical examination (CCE).

Evaluation Domain	Examinees Mean ± SD	Examiners Mean ± SD	General Quality Assessor Feedback Mean ± SD	Quality Assessor Station Observation Forms Mean ± SD
Orientation & Information	3.86 ± 1.22	4.60 ± 0.66	4.77 ± 0.49	—
Educational Value	3.94 ± 0.94	4.41 ± 0.71	—	4.68–4.78ᵃ
Logistics & Environment	3.85 ± 1.19	4.69 ± 0.59	4.74–4.91ᵇ	—
Task Clarity	3.84 ± 1.06	4.59 ± 0.67	—	—
Examiner Interaction	3.93 ± 0.99	—	—	4.77–4.80ᶜ
Candidate Comfort & Professionalism	—	4.68 ± 0.61	—	—
Simulated Patient Performance	—	—	—	4.72–4.79ᵈ
Time Allocation	—	4.54 ± 0.78	—	4.64 ± 0.68

^a^ Range of mean values across three Quality Assessor Station Observation items evaluating examination content, curriculum coverage, and higher-order clinical reasoning.

^b^ Range of mean values across Quality Assessor General Feedback items evaluating staffing, facilities, soundproofing, administration, and operational logistics.

^c^ Range of mean values across three Quality Assessor Station Observation items evaluating examiner professionalism, adherence to examination procedures, and consistency of performance across candidates.

^d^ Range of mean values across four Quality Assessor Station Observation items evaluating simulated or real patient performance; these items were completed only for stations involving simulated or real patients.

Examinees reported positive perceptions of the examination across all evaluated domains, particularly regarding its educational value and assessment of higher-order clinical reasoning. Comparatively lower ratings were observed for orientation and information, task clarity, logistics and environment, and examiner behaviour, indicating greater variability in examinee experiences across operational aspects of examination delivery.

Examiners consistently reported highly positive perceptions across all evaluated domains, with higher mean ratings and lower variability than those reported by examinees. The highest ratings were observed for examination organization, examiner support and resources, and the overall examination environment, reflecting strong confidence in the conduct and delivery of the CCE.

Quality Assessor General Feedback similarly demonstrated highly positive evaluations of examination organization, staffing, facilities, and operational processes. Quality Assessor Station Observation Forms also demonstrated consistently positive evaluations of educational value, examiner performance, simulated patient performance, and time allocation during live examination delivery.

Across all stakeholder groups, a consistent pattern emerged. While perceptions of the CCE were generally positive, examinees reported lower and more variable ratings than examiners and Quality Assessors, particularly in domains related to examination implementation. These descriptive findings provided the basis for the qualitative exploration of stakeholder experiences presented in the subsequent section.

#### 3.3.1 Orientation & information

Examinee scores were derived from the *Effectiveness of the pre-CCE orientation* item. Examiner scores represent the mean of the *Adequacy of information on CCE design and format* and *Effectiveness of pre-CCE orientation* items. Quality Assessor General Feedback represents the *Orientation and instructions provided to candidates and examiners* item.

#### 3.3.2 Educational value

Examinee scores represent the mean of the *Reflection of clinical judgment and higher-order thinking* and *Appropriateness for assessing relevant clinical skills* items. Examiner scores represent the mean of the *Assessment of clinical skills in the examination* and *Reflection of clinical judgment and critical thinking* items.

#### 3.3.3 Logistics & environment

Examinee scores were derived from the *Overall organization and logistics* item. Examiner scores were derived from the *Overall organization and flow of the CCE* item. Quality Assessor General Feedback represents operational evaluations of examination staffing, facilities, soundproofing, administration, and logistics.

#### 3.3.4 Task clarity

Examinee scores were derived from the *Clarity of instructions at each station* item. Examiner scores were derived from the *Clarity and sufficiency of instructions provided at each station* item.

#### 3.3.5 Examiner interaction

Examinee scores represent candidates' perceptions of examiner professionalism, respect, and fairness during candidate—examiner interactions. Quality Assessor Station Observation scores (ᶜ) represent independent observations of examiner performance during live examination delivery.

#### 3.3.6 Candidate comfort & professionalism

Represents examiner perceptions of candidate comfort and professionalism during the examination and is specific to the examiner questionnaire.

#### 3.3.7 Educational value

Range of mean values across the three Quality Assessor Station Observation items evaluating examination content, curriculum coverage, and assessment of higher-order clinical reasoning.

#### 3.3.8 Quality assessor general feedback

Range of mean values across items evaluating staffing, facilities, soundproofing, administration, and operational logistics.

#### 3.3.9 Examiner performance

Range of mean values across the three Quality Assessor Station Observation items evaluating examiner professionalism, adherence to examination procedures, and consistency of performance across candidates.

#### 3.3.10 Simulated patient performance

Range of mean values across the four Quality Assessor Station Observation items evaluating simulated (or real) patient performance. These items were completed only for stations involving simulated or real patients.

QA Station Observation Forms were completed by Quality Assessors following each observed candidate encounter during live examination delivery. Quality Assessors remained within individual stations for at least two consecutive candidate encounters before rotating according to a predefined schedule developed by the Specialty Examination Sub-Committee (SES). Upon submission of the final Station Observation Form, the REDCap Software platform automatically directed Quality Assessors to complete one QA General Feedback Form, providing an overall evaluation of examination organization and operational processes. During the study period, 88 Quality Assessors completed 618 QA Station Observation Forms, while 70 QA General Feedback Forms were completed. Because Station Observation Forms represent repeated operational observations contributed by the same Quality Assessors, these findings are presented descriptively rather than as independent participant-level responses.

### 3.4 Cross-stakeholder comparison

Comparison of overall perception scores demonstrated a statistically significant difference between examinees and examiners (Table 2). Because Levene's test indicated unequal variances [F(1,749) = 70.1, *p* < 0.001], Welch's independent samples t-test was interpreted. Examiners reported significantly higher overall perception scores than examinees [mean ± SD: 4.58 ± 0.53 vs 3.88 ± 0.85; Welch's t(606) = −13.4, *p* < 0.001]. The mean difference between stakeholder groups was 0.70 points on the five-point response scale, with a large effect size (Cohen's d = 0.99), indicating that the observed difference was both statistically and practically meaningful.

**Table 2:** Comparison of overall stakeholder perception scores between examinees and examiners.

Stakeholder Group	N	Mean ± SD
Examinees	367	3.88 ± 0.85
Examiners	384	4.58 ± 0.53

Statistical Test	Result	
Levene's Test	F (1,749) = 70.1, *p* < 0.001	
Welch's *t*-test	t (606) = −13.4, *p* < 0.001	
Mean Difference	−0.696	
Cohen's d	−0.985 (large effect)	

QA values are based on general feedback (one response per assessor). QA station-level data were analysed descriptively and not included in ANOVA due to repeated observations. Task clarity and examiner behaviour were not available in QA general feedback. Time allocation was analysed descriptively and not included in ANOVA due to lack of comparable data across all stakeholder groups.

### 3.5 Specialty-level perception gaps

Stakeholder responses were obtained from 19 residency and fellowship specialties, providing broad representation across postgraduate medical and dental training programme (Table 3). The largest numbers of examinee responses were contributed by Family Medicine (*n* = 91), Internal Medicine (*n* = 68), Pediatrics (*n* = 61), Emergency Medicine (*n* = 44), and Psychiatry (*n* = 33), with examiner responses demonstrating a similar distribution across specialties.

**Table 3:** Specialty-level comparison of mean overall scores across stakeholder groups (“n” represents sample size).

Specialty	Examinees n	Mean ± SD	Examiners n	Mean ± SD
Adult Rheumatology	5	3.97 ± 0.12	5	4.57 ± 0.30
Anesthesia	13	3.79 ± 0.67	8	4.49 ± 0.41
Dermatology	9	3.73 ± 0.94	13	4.72 ± 0.32
Emergency Medicine	44	4.66 ± 0.51	38	4.61 ± 0.70
Family Medicine	91	4.00 ± 0.83	106	4.58 ± 0.65
Gastroenterology	1*	4.57	5	4.38 ± 0.42
General Surgery	6	4.31 ± 0.58	16	4.55 ± 0.34
Internal Medicine	68	3.87 ± 0.73	46	4.35 ± 0.58
Neonatology	3*	4.71 ± 0.50	6	4.67 ± 0.27
Obstetrics & Gynecology	6	4.52 ± 0.69	6	4.97 ± 0.06
Orthodontics	4*	3.64 ± 0.25	6	4.45 ± 0.30
Otolaryngology–Head and Neck Surgery (ENT)	1*	4.86	7	4.76 ± 0.20
Pediatric Dentistry	8	3.63 ± 0.57	12	4.66 ± 0.42
Pediatric Endocrinology	1*	4.71	4*	4.83 ± 0.20
Pediatrics	61	3.09 ± 0.82	56	4.60 ± 0.39
Prosthodontics	4*	3.93 ± 0.88	5	4.83 ± 0.14
Psychiatry	33	3.80 ± 0.70	25	4.70 ± 0.37
Radiology	7	4.02 ± 0.21	15	4.48 ± 0.43
Urology	2*	4.00 ± 0.61	5	4.65 ± 0.58

Values are presented as mean ± standard deviation (SD).

SD could not be calculated for specialties represented by a single respondent.

*specialties with ≤5 respondents should be interpreted cautiously because estimates may be unstable. Specialty-level findings are presented descriptively and were not used for inferential comparisons.

Overall perception scores were generally positive across specialties for both examinees and examiners. Examiner ratings were consistently high across most specialties, whereas examinee ratings demonstrated greater variability. The largest differences between stakeholder groups were observed in Pediatrics, where examinees reported lower overall perception scores than examiners (mean 3.09 vs. 4.60), while several specialties, including Emergency Medicine and Neonatology, demonstrated similar overall perceptions between stakeholder groups.

Because several specialties contributed relatively small numbers of respondents (*n* ≤ 5), specialty-level findings are presented descriptively to illustrate response patterns rather than to support formal comparisons between specialties. Consequently, these findings should be interpreted cautiously, particularly for specialties represented by very small sample sizes.

## 4 Qualitative findings

### 4.1 Overview of findings

Qualitative analysis of open-ended questionnaire responses, quality assessor observations, and structured post-examination debriefing discussions identified seven overarching themes describing stakeholder experiences of the Comprehensive Clinical Examination (CCE). Across all stakeholder groups, participants consistently described the examination as well organized, educationally valuable, and clinically relevant. At the same time, they highlighted areas of variability related to examination implementation, including task clarity, time allocation, examiner interactions, simulated patient performance, and operational processes.

Although the overall direction of stakeholder feedback was broadly consistent, each stakeholder group contributed distinct perspectives. Examinees primarily described their lived examination experiences, examiners focused on assessment delivery and educational value, while quality assessors identified operational issues observed during live examination delivery. Together, these complementary perspectives provided a comprehensive understanding of the strengths of the examination and opportunities for quality improvement.

The seven themes identified through thematic analysis are summarized in Table 4 and presented below with representative quotations from participants.

**Table 4:** Summary of themes and sub-themes.

Theme	Sub-themes
Exam Organization and Logistical Management	Organization and coordination, administrative processes, facilities, scheduling, and logistical support
Station Design, Content, and Blueprint Alignment	Clinical relevance, difficulty level, alignment with training
Clarity of Task Expectations and Instructions	Instruction clarity, consistency, pre-exam orientation
Time Allocation, Flow, and Cognitive Load	Waiting time, station timing, workload, flow
Examiner Performance and Behaviour	Professionalism, prompting, consistency, communication
Simulated Patient (SP) Performance and Consistency	Communication clarity, realism, consistency
Assessment Fairness, Scoring, and Psychological Impact	Scoring consistency, fairness perception, stress

### 4.2 Theme 1: exam organization and logistical management

Stakeholders across all three groups generally described the CCE as a well-organized examination with effective coordination, clear administrative processes, and supportive examination staff. Positive comments frequently highlighted the smooth conduct of the examination, appropriate facilities, and efficient logistical arrangements.

“Very well-organized exam… smooth process.” Examinee (Internal Medicine, Comment 225).

“Facilities are excellent with supportive staff.” QA (Psychiatry, General Feedback Comment 14).

“Good experience, well-organized communication, circuits, registration, and timings.” (Family Medicine Examinee, Focus Group).

Despite these positive perceptions, participants also identified variability in aspects of examination logistics. Examinees commonly reported prolonged waiting periods before entering the examination and extended examination days, which were perceived to contribute to fatigue and reduced concentration.

Operational observations recorded by quality assessors and examiner comments also identified occasional environmental issues, including noise and venue-related factors that affected examination flow.

“Periods of noise due to construction.” (Family Medicine Examiner Comment 62).

### 4.3 Theme 2: station design, content, and blueprint alignment

Stakeholders across all groups generally perceived the examination stations as clinically relevant and aligned with specialist practice. Examinees, examiners, and quality assessors frequently described the stations as reflecting authentic clinical scenarios and appropriately assessing higher-order clinical reasoning and decision-making.

“Stations were clinically relevant and mimicked real-life scenarios.” (General Surgery Examinee, Comment 30).

“Well-written station assessing different aspects of the curriculum.” (Psychiatry QA, Comment 14).

Despite these positive perceptions, some participants identified variation in the perceived relevance, level of difficulty, and alignment of individual stations with routine clinical practice. These comments suggested that experiences differed across specialties and examination stations rather than reflecting concerns about the examination as a whole.

“Some stations were not reflective of daily practice.” (Pediatrics Examinee, Comment 129).

“Questions did not evaluate decision-making process.” (General Surgery QA, Comment 69).

### 4.4 Theme 3: clarity of task expectations and instructions

Stakeholder feedback indicated that many examination stations provided clear instructions and well-defined task expectations. Participants frequently described written and verbal instructions as sufficiently detailed to guide candidate performance.

“Instructions were clear for both examiners and examinee s.” (Emergency Medicine Examinee, Comment 253).

At the same time, participants described inconsistency in the clarity of instructions across some stations. Examinees reported uncertainty regarding expected responses, while quality assessors observed occasions where written instructions required further clarification.

“Tasks were unclear… difficult to know what was expected.” (Psychiatry Examinee, Comment 43).

“Some stations the scenario was not clear what was expected from us.” (Internal Medicine Examinee, Comment 8).

Quality assessor observations reflected similar concerns.

“Instructions… are confusing for examinee s.” (Internal Medicine QA, Comment 1).

### 4.5 Theme 4: time allocation, flow, and cognitive load

Stakeholders generally considered the allocated station time to be appropriate for completion of examination tasks. Quality assessors frequently reported that most stations allowed sufficient time for candidates to complete the required activities.

“Time allocated for the station was adequate.” (Family Medicine QA, Comment 94).

Nevertheless, participants also reported that time adequacy differed across some stations. Examinees frequently described prolonged waiting periods before the examination and perceived time pressure during selected stations, while quality assessors observed occasions where task requirements appeared difficult to complete within the allocated time.

“Too many points to cover… felt rushed.” (Internal Medicine QA, Comment 1).

“Not possible to complete all tasks in time.” (Family Medicine Examinee Comment 353).

“Some stations are too tight for time.” (General Surgery Examinee, Focus Group).

“Only too much waiting as half the exam was in the morning and half in the evening.” (Psychiatry Examinee, Focus Group).

“We spent almost 10 h in the center… which is taxing.” (Psychiatry, Examinee Comment 40).

### 4.6 Theme 5: examiner performance and behaviour

Participants consistently described examiner conduct as professional, respectful, and supportive. Positive comments emphasized courteous interactions and professional behaviour throughout the examination.

“Examiners were professional and respectful.” (Emergency Medicine Examinee, Comment 240).

“Maintained eye contact… courteous.” (General Surgery, QA Comment 13).

Alongside these positive perceptions, participants also described differences in examiner communication styles and prompting practices across some stations. These observations were reported primarily by examinees and were also documented by quality assessors.

“Some examiners were aggressive.” (Pediatrics Examinee Comment 125).

“The examiner kept saying “are you sure?’ which increased stress.” (Pediatrics Examinee, Comment 133).

Quality assessor observations similarly noted occasional differences in prompting approaches.

“Comments given from examiners are appropriate but sometimes leading.” (Family Medicine QA, Comment 17).

### 4.7 Theme 6: simulated patient (SP) performance and consistency

Stakeholders generally described simulated patient (SP) performance as realistic, professional, and supportive of authentic clinical assessment. Examiners and quality assessors frequently highlighted the contribution of well-trained SPs to the overall quality of the examination.

“SP was very good… fair and consistent.” (Pediatrics Examiner, Comment 122).

“Excellent SP performance.” (Family Medicine QA, Comment 20).

Despite these positive observations, some participants reported differences in SP communication and consistency between candidate encounters. These comments were primarily related to variability in responses provided by SPs across different candidates rather than concerns regarding the overall quality of SP performance.

“SP was not consistent across examinee s.” (Internal Medicine Examiner, Comment 211).

“SP responses were unclear.” (Psychiatry Examinee, Comment 33).

### 4.8 Theme 7: assessment fairness, scoring, and examinee experience

Participants generally perceived the examination as fair and acceptable, with many describing the assessment as appropriate for evaluating specialist-level clinical competence.

“The exam was fair and acceptable.” (Internal Medicine Examinee Comment 22).

At the same time, some participants described differences in examiner interactions and scoring practices across stations that they believed influenced their examination experience. Quality assessors also documented occasional differences in assessment practices during live examination observations.

“Marks depended on examiner attitude.” (Family Medicine Examinee, Comment 116).

“Different Examinees were asked different questions.” (General Surgery Examinee, Comment 31).

Quality assessor observations similarly identified occasional differences in assessment practices.

“Examiners discussed assessments.” (Internal Medicine QA, Comment 1).

In addition to comments regarding assessment processes, examinees frequently described emotional and physical experiences associated with participating in a high-stakes examination, including stress, fatigue, uncertainty, and prolonged waiting times.

“Waiting and stress affected performance.” (Pediatrics Examinee, Comment 130).

“Waiting for 5 h before the exam made us exhausted.” (Pediatrics, Examinee Comment 130).

“Long waiting time… affected concentration.” (Psychiatry Examinee, Comment 50).

Consistent with the convergent mixed-methods design (16, 17), integration of quantitative and qualitative findings occurred during the interpretation stage using a triangulation approach. Quantitative results were compared with qualitative themes to identify areas of convergence, complementarity, and divergence across stakeholder groups. This integrated interpretation enabled quantitative patterns to be explained and contextualized by participants' experiences, providing a more comprehensive understanding of examination implementation and stakeholder perceptions than either dataset could provide independently.

## 5 Discussion

This mixed-methods, multi-stakeholder evaluation provides a comprehensive assessment of stakeholder perceptions of the Emirati Board Comprehensive Clinical Examination (CCE), a national high-stakes postgraduate specialist certification examination in the United Arab Emirates. Consistent with the convergent mixed-methods design, integration of the quantitative and qualitative findings during interpretation demonstrated substantial convergence across stakeholder groups while also identifying important areas of complementarity. Across both quantitative and qualitative findings, stakeholders consistently perceived the examination as well organized, educationally valuable, and clinically relevant. Nevertheless, important differences emerged between stakeholder groups. Examinees reported lower and more variable perceptions than examiners and Quality Assessors, particularly regarding orientation, task clarity, logistics, and aspects of examination implementation. Qualitative findings complemented these observations by identifying inconsistencies in task instructions, examiner interactions, time allocation, and simulated patient performance across some examination stations. Together, these findings suggest that while the overall examination process was viewed positively, stakeholders experienced operational aspects of examination delivery differently.

The difference between examinee and examiner perceptions is not unexpected and has been reported previously in evaluations of OSCEs and other high-stakes clinical assessments (3, 6, 8–14). Examinees experience the assessment directly and are therefore more sensitive to operational issues such as waiting times, clarity of instructions, examiner communication, and station-specific implementation. In contrast, examiners focus primarily on assessment delivery and candidate performance, whereas Quality Assessors observe examination implementation across multiple stations from an independent quality assurance perspective. Collectively, these complementary perspectives provide a broader understanding of examination quality than would be achieved through evaluation of any single stakeholder group alone.

Although overall perception scores demonstrated some variation across specialties, the general pattern of positive stakeholder perceptions was observed across a broad range of residency and fellowship programmes. The specialty-level analysis was undertaken primarily to demonstrate that the evaluation framework was applied across multiple disciplines rather than to compare specialties directly. Because several specialties contributed relatively small numbers of respondents, these findings were interpreted descriptively and should not be viewed as evidence of differences in examination quality between specialty programmes.

The present findings are consistent with previous studies demonstrating that well-designed OSCE-style examinations are generally perceived as educationally valuable while operational aspects of examination delivery continue to influence stakeholder experiences (2, 3, 6–11). Similar observations have been reported in studies highlighting the importance of examiner preparation, orientation, station standardization, and quality assurance processes in supporting high-quality clinical assessment (3, 8, 9, 11). Within the regional context, Ibrahim et al. (14) similarly highlighted examination-related factors influencing stakeholder experiences during a national licensure OSCE in the United Arab Emirates. Unlike previous studies that focused primarily on psychometric performance or a single stakeholder group, the present study integrated the perspectives of examinees, examiners, and Quality Assessors together with structured operational observations collected during live examination delivery. This multi-stakeholder approach provided complementary evidence regarding examination implementation and quality assurance.

Qualitative findings further enriched the quantitative results by providing contextual explanations for the observed perception patterns. While quantitative findings demonstrated generally positive perceptions across stakeholder groups, qualitative comments highlighted operational issues including occasional variation in task clarity, examiner interactions, simulated patient consistency, waiting times, and time allocation. These observations should be interpreted as stakeholder perceptions and operational observations rather than direct evidence of examination validity or reliability. Nevertheless, they identify practical opportunities for strengthening consistency of examination implementation through continuous quality improvement.

Together, these findings demonstrate how integration of quantitative and qualitative evidence provides a more comprehensive understanding of examination implementation than either dataset could provide independently. A joint display summarizing the integration of quantitative and qualitative findings across the principal examination domains is provided in Supplementary Table S10.

An important contribution of this study is the description of the governance framework supporting the Emirati Board CCE. Rather than relying solely on post-examination surveys, the NIHS quality assurance system integrates prospective Quality Assessor observations, structured stakeholder feedback, post-examination debriefing discussions, and formal Quality Committee review within a continuous quality improvement cycle. This governance approach enables operational findings identified during examination delivery to be translated into structured action plans that are monitored during subsequent examination cycles. Such a model may provide a practical framework for other postgraduate assessment programmes seeking to strengthen quality assurance within high-stakes clinical examinations, consistent with recommendations promoting collaborative approaches to improving assessment practice across institutions (15).

Although this study was not designed to establish psychometric validity or examination reliability directly, the findings provide perception-based evidence regarding examination implementation that may inform future evaluation of assessment quality. Contemporary assessment frameworks emphasize that assessment quality depends not only on examination design but also on the consistency with which assessment processes are implemented (25–27). Future studies integrating stakeholder perceptions with psychometric evidence, examiner agreement, and candidate performance outcomes would provide a more comprehensive understanding of the relationship between examination implementation and assessment quality.

## 6 Strengths of the study

This study has several important strengths. First, it represents one of the first comprehensive multi-stakeholder evaluations of a national postgraduate specialist certification examination conducted within the United Arab Emirates. By incorporating the perspectives of examinees, examiners, and Quality Assessors, the study provides a broader evaluation of examination quality than studies relying on a single stakeholder group.

Second, the study was conducted within the National Institute for Health Specialties' established governance and quality assurance framework. In addition to post-examination stakeholder surveys, the evaluation incorporated prospective Quality Assessor observations undertaken during live examination delivery together with structured post-examination debriefing discussions. These complementary data sources strengthened the interpretation of findings by providing both participant perspectives and independent operational observations.

Third, the study employed a convergent mixed-methods design, integrating quantitative and qualitative findings to provide a comprehensive understanding of stakeholder experiences. This approach enabled quantitative perception patterns to be contextualized through qualitative feedback, thereby identifying practical opportunities for quality improvement that may not have been evident from survey data alone.

Finally, the findings are directly applicable to routine examination governance. By integrating stakeholder feedback, operational observations, and Quality Committee review within an established continuous quality improvement cycle, the study demonstrates a practical framework that may be transferable to other high-stakes postgraduate clinical assessment programmes.

## 7 Limitations

Several limitations should be considered when interpreting the findings. First, this study evaluated stakeholder perceptions and operational observations rather than psychometric characteristics, examiner agreement, or candidate performance outcomes. Accordingly, the findings should not be interpreted as direct evidence of examination validity or reliability but rather as perception-based evidence informing examination quality and continuous improvement.

Second, Quality Assessor Station Observation Forms comprised repeated observations completed by the same Quality Assessors across multiple candidate encounters. Although these observations provided valuable insight into examination implementation, they were analysed descriptively and should not be interpreted as statistically independent participant-level responses.

Third, responses were pooled across multiple examination sittings to provide an overall evaluation of examination implementation. Consequently, the study was not designed to evaluate temporal changes or the impact of quality improvement initiatives across successive examination cycles.

Fourth, several specialties contributed relatively small numbers of respondents. Specialty-level findings were therefore presented descriptively and should be interpreted cautiously, particularly for specialties represented by limited sample sizes.

Finally, although response rates were high across stakeholder groups, the possibility of non-response bias cannot be excluded because individuals who chose not to participate may have held different views from respondents. Because completion of the evaluation formed part of the routine examination quality assurance programme, some participants may also have perceived an expectation to participate despite the voluntary electronic consent for research use of their data. Social desirability bias may have influenced responses, particularly among examiners and Quality Assessors, although anonymous questionnaires for examinees and examiners were used to encourage honest feedback. Furthermore, qualitative findings were derived from questionnaire comments and contemporaneously documented field notes from structured post-examination debriefing discussions rather than verbatim interview transcripts, which may have limited the depth of participants' perspectives. Potential institutional bias arising from researcher involvement in the examination process was addressed through the reflexivity procedures described in Section 4.6.1. Future research integrating stakeholder perceptions with psychometric indicators, examiner agreement, and candidate performance outcomes would provide a more comprehensive evaluation of examination quality in high-stakes clinical assessment.

## 8 Conclusions

This mixed-methods, multi-stakeholder evaluation demonstrated that the Emirati Board Comprehensive Clinical Examination is generally perceived as a well-organized, educationally valuable, and clinically relevant high-stakes assessment. Although stakeholders consistently reported positive perceptions of the examination, examinees experienced greater variability than examiners and Quality Assessors in operational aspects of examination delivery, particularly regarding orientation, logistics, task clarity, and examiner interactions.

The integration of stakeholder feedback with prospective Quality Assessor observations within a structured governance framework provides valuable evidence to support continuous quality improvement of high-stakes clinical examinations. Rather than indicating deficiencies in examination design, the findings identify opportunities to enhance the consistency of examination implementation through structured examiner preparation, calibration, quality assurance monitoring, and systematic stakeholder feedback.

Future studies integrating stakeholder perceptions with psychometric evidence, examiner agreement, and candidate performance outcomes will further strengthen understanding of how examination implementation influences assessment quality and support the ongoing enhancement of postgraduate specialist certification examinations.

## Data Availability

The datasets generated and analysed during the current study are not publicly available because they contain examination quality assurance data governed by the National Institute for Health Specialties (NIHS). De-identified data may be made available from the corresponding author upon reasonable request, subject to institutional approval and applicable examination governance and ethical requirements.

## Supplementary material

The Supplementary Material for this article can be found online at: https://www.frontiersin.org/articles/10.3389/fmed.2026.1900166/full#supplementary-material

Supplementary Table S1 COREQ Checklist.

Supplementary Table S2 Qualitative Codebook.

Supplementary Table S3 Additional Representative Participant Quotations.

Supplementary Table S4 Stakeholder Questionnaires.

Supplementary Table S5 Internal consistency reliability analyses of the study instruments.

Supplementary Document Ethics approval.

Supplementary Table S6-S9 Item-level descriptive statistics for examinee, examiner, and Quality Assessor questionnaires.

Supplementary Table S10 Joint Display of Integrated Quantitative and Qualitative Findings Across Major Examination Domains

Supplementary Document S11 AI prompts and workflow used for organization of qualitative data.
