# Supplementary material for: Stakeholder perspectives on the Emirati board comprehensive clinical examination: a mixed-methods multi-stakeholder evaluation
Source: Front Med (Lausanne). 2026 Oct 14;13:1900166. doi: 10.3389/fmed.2026.1900166 (PMC13713724; doi:10.3389/fmed.2026.1900166)
Supplement: Supplementary file 1 [file Datasheet1.pdf]

COREQ (COnsolidated criteria for REporting Qualitative research) Checklist

A checklist of items that should be included in reports of qualitative research. You must report the page number in your manuscript where you consider each of the items listed in this checklist. If you have not included this information, either revise your manuscript accordingly before submitting or note N/A.

Topic	Item No.	Guide Questions/Description	Reported on Page No.
**Domain 1: Research team and reflexivity**			
Personal characteristics			
Interviewer/facilitator	1	Which author/s conducted the interview or focus group?	Dr. Asma / Dr. Fouzia / Dr. Ibrahim / Dr. Hadeel<br>The study team conducted data collection through surveys and facilitated debriefing sessions.
Credentials	2	What were the researcher's credentials? E.g. PhD, MD	The research team comprised clinicians and medical education researchers. PhD/MD
Occupation	3	What was their occupation at the time of the study?	Researchers were involved in postgraduate medical education and Board Examinations
Gender	4	Was the researcher male or female?	Male & Female
Experience and training	5	What experience or training did the researcher have?	The research team comprised experienced medical education researchers with prior publications in assessment and qualitative research.
Relationship with participants			
Relationship established	6	Was a relationship established prior to study commencement?	No direct researcher participant relationship was established specifically for the purposes of this study. Data collection was conducted anonymously through electronic surveys, and participation was voluntary, minimizing the potential influence of prior professional relationships.
Participant knowledge of the interviewer	7	What did the participants know about the researcher? e.g. personal goals, reasons for doing the research	Participants were aware that feedback was being collected for quality improvement purposes; however, no prior personal relationship existed between researchers and participants.
Interviewer characteristics	8	What characteristics were reported about the inter viewer/facilitator? e.g. Bias, assumptions, reasons and interests in the research topic	Researchers had expertise in medical education and assessment, which informed study design and analysis.
**Domain 2: Study design**			
Theoretical framework			
Methodological orientation and Theory	9	What methodological orientation was stated to underpin the study? e.g. grounded theory, discourse analysis, ethnography, phenomenology, content analysis	The qualitative component was underpinned by an interpretivist methodological orientation, employing inductive reflexive thematic analysis as described by Braun and Clarke.
Participant selection			
Sampling	10	How were participants selected? e.g. purposive, convenience, consecutive, snowball	Participants were selected using a combination of convenience and purposive sampling.
Method of approach	11	How were participants approached? e.g. face-to-face, telephone, mail, email	Participants were approached via email and online survey distribution; debriefing participants were approached face-to-face post-examination.
Sample size	12	How many participants were in the study?	368 examinees, 390 examiners, and 88 quality assessors.
Non-participation	13	How many people refused to participate or dropped out? Reasons?	Response rates were calculated for all stakeholder groups and reported as part of routine reports to examination quality committee. However, individual-level data on refusal to participate or reasons for non-participation were not formally collected, as survey participation was voluntary and anonymous. No dropouts were applicable for debriefing sessions, as participation was completed at a single time point.
Setting			
Setting of data collection	14	Where was the data collected? e.g. home, clinic, workplace	Quantitative data were collected electronically; qualitative data were collected during on-site debriefing sessions.
Presence of non-participants	15	Was anyone else present besides the participants and researchers?	Only participants and facilitators were present during debriefing sessions.
Description of sample	16	What are the important characteristics of the sample? e.g. demographic data, date	Participants included postgraduate trainees, examiners, and QA assessors across multiple specialties from Emirati Board CCE cycles (2024–2026).
Data collection			
Interview guide	17	Were questions, prompts, guides provided by the authors? Was it pilot tested?	Yes. Structured questionnaires and open-ended prompts were developed based on predefined domains and reviewed by subject matter experts.

Repeat interviews	18	Were repeat interviews carried out? If yes, how many?	No repeat interviews were conducted.
Audio/visual recording	19	Did the research use audio or visual recording to collect the data?	No recordings were used.
Field notes	20	Were field notes made during and/or after the interview or focus group?	Yes. Field notes were recorded during debriefing sessions
Duration	21	What was the duration of the inter views or focus group?	Debriefing sessions lasted approximately 20–40 minutes.
Data saturation	22	Was data saturation discussed?	Data saturation was not formally assessed, as the study aimed to capture comprehensive stakeholder feedback rather than achieve thematic saturation.
Transcripts returned	23	Were transcripts returned to participants for comment and/or correction?	Not applicable; no transcripts were generated.
**Domain 3: analysis and findings**			
Data analysis			
Number of data coders	24	How many data coders coded the data?	Four researchers contributed to coding and theme development.
Description of the coding tree	25	Did authors provide a description of the coding tree?	Four researchers contributed to coding and theme development.
Derivation of themes	26	Were themes identified in advance or derived from the data?	Themes were derived inductively from the data.
Software	27	What software, if applicable, was used to manage the data?	Quantitative data were analysed using Python (pandas, SciPy, statsmodels). Qualitative data were managed using structured coding frameworks and audit trails.
Participant checking	28	Did participants provide feedback on the findings?	Formal member checking was not conducted. However, findings informed quality improvement processes through reporting to the Examination Sub-Committee.
Reporting			
Quotations presented	29	Were participant quotations presented to illustrate the themes/findings? Was each quotation identified? e.g. participant number	Yes. Verbatim quotations were used and identified by stakeholder group and source.
Data and findings consistent	30	Was there consistency between the data presented and the findings?	Yes. Findings were grounded in the data.
Clarity of major themes	31	Were major themes clearly presented in the findings?	Yes. Major themes were clearly presented.
Clarity of minor themes	32	Is there a description of diverse cases or discussion of minor themes?	Yes. Variability and diverse perspectives were described across stakeholder groups.

Developed from: Tong A, Sainsbury P, Craig J. Consolidated criteria for reporting qualitative research (COREQ): a 32-item checklist for interviews and focus groups. *International Journal for Quality in Health Care*. 2007. Volume 19, Number 6: pp. 349 – 357

Once you have completed this checklist, please save a copy and upload it as part of your submission. DO NOT include this checklist as part of the main manuscript document. It must be uploaded as a separate file.
