# Supplementary material for: Stakeholder perspectives on the Emirati board comprehensive clinical examination: a mixed-methods multi-stakeholder evaluation
Source: Front Med (Lausanne). 2026 Oct 14;13:1900166. doi: 10.3389/fmed.2026.1900166 (PMC13713724; doi:10.3389/fmed.2026.1900166)
Supplement: Supplementary file 2 [file Datasheet2.pdf]

The codebook was developed iteratively and refined through team consensus during the coding process			
**Code**	**Code Description**	**Sub-theme**	**Theme**
Organization quality	Overall structure, coordination, smooth flo	Organization and coordinatior	Exam Organization and Logistical Management
Examinee flow issues/Scheduling D	Delays, scheduling problems, group timing	Examinee flow across stations	Exam Organization and Logistical Management
Environmental conditions	Noise, temperature, physical setup issues	Environmental conditions	Exam Organization and Logistical Management
Technical/logistical support	IT, devices, equipment, staff support	Logistics and support	Exam Organization and Logistical Management
Clinical relevance	Alignment of cases with real-world practice	Clinical relevance	Station Design, Content, and Blueprint Alignment
Difficulty Level	Perceived level of difficulty (too easy/too c	Difficulty level	Station Design, Content, and Blueprint Alignment
Alignment with training	Relevance to trainee level and curriculum	Alignment with training	Station Design, Content, and Blueprint Alignment
Case complexity	Multiple tasks, excessive breadth within st	Task complexity	Station Design, Content, and Blueprint Alignment
Instruction clarity	Clarity of written instructions	Clarity of instructions	Clarity of Task Expectations and Instructions
Verbal instruction consistency	Differences between examiner instructions	Consistency of instructions	Clarity of Task Expectations and Instructions
Task expectation ambiguity	Unclear what is required in station	Task expectations	Clarity of Task Expectations and Instructions
Pre-exam orientation	Adequacy of briefing and preparation	Orientation	Clarity of Task Expectations and Instructions
Waiting time	Pre-exam delays and waiting periods	Pre-exam waiting	Time Allocation, Flow, and Cognitive Load
Station time adequacy	Time sufficient/insufficient per station	Within-station timing	Time Allocation, Flow, and Cognitive Load
Workload imbalance	Too many tasks within limited time	Task complexity	Time Allocation, Flow, and Cognitive Load
Cognitive load	Stress, overload, difficulty processing task	Cognitive load	Time Allocation, Flow, and Cognitive Load
Examiner professionalism	Respect, neutrality, communication style	Professionalism	Examiner Performance and Behaviour
Examiner prompting	Guidance, prompting, leading questions	Prompting and guidance	Examiner Performance and Behaviour
Examiner variability	Differences across examiners	Consistency	Examiner Performance and Behaviour
Examiner interaction tone	Supportive vs intimidating behaviour	Communication style	Examiner Performance and Behaviour
SP realism	Authenticity of SP performance	Realism	Simulated Patient Performance and Consistency
SP communication clarity	Clear vs confusing responses	Communication clarity	Simulated Patient Performance and Consistency
SP consistency	Variation in performance between Examin	Consistency	Simulated Patient Performance and Consistency
SP training issues	Lack of standardization or preparation	Training	Simulated Patient Performance and Consistency
Fairness perception	Perceived fairness of exam	Fairness	Assessment Fairness, Scoring, and Psychological Impact
Scoring consistency	Variability in marking	Scoring consistency	Assessment Fairness, Scoring, and Psychological Impact
Examiner bias perception	Perceived influence of examiner behaviou	Bias	Assessment Fairness, Scoring, and Psychological Impact
Psychological stress	Anxiety, fatigue, emotional impact	Psychological experience	Assessment Fairness, Scoring, and Psychological Impact
Performance impact	Effect of stress/logistics on performance	Performance impact	Assessment Fairness, Scoring, and Psychological Impact
