# Supplementary material for: Stakeholder perspectives on the Emirati board comprehensive clinical examination: a mixed-methods multi-stakeholder evaluation
Source: Front Med (Lausanne). 2026 Oct 14;13:1900166. doi: 10.3389/fmed.2026.1900166 (PMC13713724; doi:10.3389/fmed.2026.1900166)
Supplement: Supplementary file 3 [file Datasheet3.pdf]

Theme	Sub-theme	Stakeholder	Quotation		
Exam Organization	Scheduling	Examinee	We waited for several hours before starting, which affected our focus.		
Exam Organization	Environment	Examiner	There were occasional disturbances due to noise.		
Station Design	Relevance	Examinee	Some stations did not reflect real clinical practice.		
Station Design	Difficulty	QA	Questions were too basic and did not assess decision-making.		
Task Clarity	Instructions	Examinee	It was difficult to understand what was expected.		
Task Clarity	Consistency	QA	Instructions were not always consistent across stations.		
Time Allocation	Waiting	Examinee	The long waiting time was exhausting.		
Time Allocation	Workload	QA	Too many tasks to complete within the station time.		
Examiner Behaviour	Professionalism	Examinee	Examiners were respectful and professional.		
Examiner Behaviour	Prompting	Examinee	Some examiners kept questioning in a stressful way.		
SP Performance	Realism	Examiner	The simulated patient was realistic and consistent.		
SP Performance	Consistency	Examinee	SP responses varied between examinees.		
Fairness	Scoring	Examinee	“Marks seemed more dependent on the examiner’s attitude than on the candidate’s actual performance”		
Fairness	Psychological impact	Examinee	Stress and fatigue affected my performance.		
