# Supplementary material for: Stakeholder perspectives on the Emirati board comprehensive clinical examination: a mixed-methods multi-stakeholder evaluation
Source: Front Med (Lausanne). 2026 Oct 14;13:1900166. doi: 10.3389/fmed.2026.1900166 (PMC13713724; doi:10.3389/fmed.2026.1900166)
Supplement: Supplementary file 4 [file Datasheet4.pdf]

Examiner Survey - Clinical Comprehensive Examination Survey

Dear Examiner,Greetings!We request your feedback on the Clinical Comprehensive Examination (CCE). As an examiner, your insights are essential to help us assess and improve the examination's quality, relevance, and effectiveness.The objective of the survey is to gather your feedback on the following aspects: - Examination Format - Structure and clarity - Technical Content - Appropriateness and comprehensiveness - Delivery & Organization - Smoothness of execution and setup - Management & Administration - Effectiveness of support and communicationYour input will be invaluable in our efforts to maintain the highest standards and align with global best practices. The survey takes approximately 3-5 minutes, and all responses will remain confidential.Thank you!

---

1) I give my consent that this data can be used for NIHS Quality Improvement and Research Purposes.
- ◯ Yes
- ◯ No

Please select the appropriate station details
- Speciality Name ______
- Station Number ______
- Circuit Number ______

Exam Date ______

---

2) Select the exam day for which feedback is being provided
- ◯ Feedback of DAY 1
- ◯ Feedback of DAY 2

(Day 2 applies when exam is scheduled over 2 days)

---

3) Please select the examination period
- ◯ Jan-Feb 2026
- ◯ Nov-Dec 2025
- ◯ Nov - Dec, 2024
- ◯ Mar - Apr 2025
- ◯ May - June 2025
- ◯ July -August 2025
- ◯ Sept-Oct 2025

Please provide your comments related to your experience with
- Assessment content and examination standard ______
- Examination administration and communication ______

Station specific feedback ______

My experience with the following statements related to the Clinical Comprehensive Examination (CCE) was satisfactory

	Strongly Agree	Agree	Neutral	Disagree	Strongly Disagree
4) Adequacy of Information on CCE design and format	◯	◯	◯	◯	◯
5) Effectiveness of Pre-CCE Orientation	◯	◯	◯	◯	◯
6) Assessment of Clinical Skills in the exam	◯	◯	◯	◯	◯
7) Reflection of Clinical Judgment and Critical Thinking on exam stations	◯	◯	◯	◯	◯
8) Efficiency and Usability of the Electronic Scoring System	◯	◯	◯	◯	◯

9)

	Clarity and Sufficiency of Instructions Provided at Each Station	○	○	○	○	○
10)	Overall Organization and Flow of the CCE	○	○	○	○	○
11)	Adequacy of Examiner Support and Resources Provided	○	○	○	○	○
12)	Soundproofing and Privacy at Each Exam Station	○	○	○	○	○
13)	Candidate Comfort and Professionalism During the Exam	○	○	○	○	○
14)	Time Allocation per Station	○	○	○	○	○
15)	Clarity of Assessment Criteria and Scoring Rubrics	○	○	○	○	○
16)	Examiner Autonomy in Providing Feedback and Guidance	○	○	○	○	○

Invitation to Provide Feedback on the Clinical Comprehensive Examination (CCE)

Dear Examinee,Greetings!We invite you to participate in an important survey to gather feedback on your experience with the Clinical Comprehensive Examination (CCE). Your insights are crucial in helping us assess and enhance the quality and effectiveness of the CCE, ensuring it meets the highest standards of competency assessment.This survey will ask for your feedback on various aspects of the CCE, including: - Examination Format - Clarity, relevance, and suitability of the structure. - Technical Content - Appropriateness and comprehensiveness of the knowledge and skills tested. - Delivery, Execution, and Organization - Effectiveness of the examination's setup, scheduling, and overall flow. - Management and Administration - Quality of logistical support, communication, and general administration.Your honest feedback will directly contribute to our ongoing efforts to uphold and improve the quality of the examination. We aim to align with global best practices to deliver an assessment experience that is rigorous, fair, and supportive of your professional development.The survey should take approximately 3 minutes to complete. All responses will be kept confidential and used solely for quality improvement purposes.

---

1) I give my consent that this data can be used for NIHS Quality Improvement and Research Purposes.

◯ Yes
◯ No

---

2) Please select the examination date

____

---

3) Please select the examination period

◯ Nov-Dec 2025
◯ Jan-Feb 2026
◯ Mar-April 2026
◯ May-June 2026
◯ Nov - Dec 2024
◯ Mar - Apr 2025
◯ May - June 2025
◯ Sept-Oct 2025

---

4) Please select your specialty

◯ Internal Medicine
◯ General Surgery
◯ Psychiatry
◯ Family Medicine
◯ Dermatology
◯ Obstetrics & Gynecology
◯ Pediatrics
◯ Otolaryngology Head and Neck Surgery (ENT)
◯ Anesthesia
◯ Emergency Medicine
◯ Pediatric Endocrinology
◯ Gasteroenterology
◯ Adult Rheumatology
◯ Pediatric Dentistry
◯ Prosthodontics
◯ Orthodontics
◯ Neonatology
◯ Radiology
◯ Urology

My experience with the following statements related to the Clinical Comprehensive Examination (CCE) was satisfactory

		Strongly Agree	Agree	Neutral	Disagree	Strongly Disagree
5)	Examiner interaction at my station: The examiner(s) were professional, respectful, and conducted a fair, unbiased interaction	◯	◯	◯	◯	◯
6)	Adequacy of station information/design: The station provided enough relevant information to answer the questions (case, data/images)	◯	◯	◯	◯	◯
7)	Effectiveness of the pre-CCE orientation: The briefing prepared me for the format, rules, timing, marking approach, and what to expect at each station.	◯	◯	◯	◯	◯
8)	Appropriateness for assessing relevant clinical skills: The content appropriately assessed targeted competencies	◯	◯	◯	◯	◯
9)	Reflection of clinical judgment & higher-order thinking: The station required application of clinical judgment (prioritization, risk stratification, decision-making) rather than recall alone.	◯	◯	◯	◯	◯
10)	Clarity of instructions at each station: Instructions were clear, specific, and unambiguous (tasks, time limits, and expected outputs were easy to understand).	◯	◯	◯	◯	◯
11)	Overall organization & logistics: Scheduling, rotation flow, venue setup, materials/technology, and allotted time supported a smooth examination experience.	◯	◯	◯	◯	◯

12) Please provide comments on areas of strengths and constructive feedback for areas needing improvement

____

Quality Assessor's Station-wise Feedback - Clinical Comprehensive Examination Survey

Collect feedback separately for each station.After finishing one station, click "Evaluate next station" to proceed.Click "Submit" only after all stations are evaluated

Please mention the station details
Station Number : ______ Round : ______ Circuit : ______
1st Examiner Name : ______
2nd Examiner Name : ______

Examination Content

To what extent,

		Excellent	Good	Neutral	Poor	Very Poor	Not Applicable
1)	Does the content adequately cover a range of knowledge and skills?	◯	◯	◯	◯	◯	◯
2)	Does the content sample across the curriculum without excessive repetition?	◯	◯	◯	◯	◯	◯
3)	Does the exam avoid excessive factual recall and encourage higher-order thinking?	◯	◯	◯	◯	◯	◯
4)	Is time allocation adequate for task completion?	◯	◯	◯	◯	◯	◯

Candidate Preparation

To what extent,

		Excellent	Good	Neutral	Poor	Very Poor
5)	Does the candidate communicate effectively?	◯	◯	◯	◯	◯
6)	Does the candidate present themselves professionally?	◯	◯	◯	◯	◯
7)	Are the candidates well-prepared for this exam?	◯	◯	◯	◯	◯

Standardized Patient (SP)/Real Patient Performance

To what extent,

		Excellent	Good	Neutral	Poor	Very Poor	Not Applicable
8)	Is SP/real patient provided with adequate guidance?	◯	◯	◯	◯	◯	◯
9)	Is the patient comfortable?	◯	◯	◯	◯	◯	◯
10)	Is the patient well-trained and competent?	◯	◯	◯	◯	◯	◯
11)	Is the SP performance standardized and consistent between various candidate encounters?	◯	◯	◯	◯	◯	◯

Examiner Performance

To what extent,

		Excellent	Good	Neutral	Poor	Very Poor	Not Applicable
12)	Does the examiner adhere to the script?	◯	◯	◯	◯	◯	◯
13)	Is the examiner courteous and polite?	◯	◯	◯	◯	◯	◯
14)	Is the examiner, standardized and consistent between various candidate encounters?	◯	◯	◯	◯	◯	◯

15) Please provide comments on Assessment Content and Examination Standard

____

General Feedback about your overall experience with CCE!!

Feedback on CCE Preparations

To what extent,	Excellent	Good	Neutral	Poor	Very Poor
1) Orientation and instructions provided to candidates and examiners were effective?	○	○	○	○	○
2) All necessary material before the start of the exam was well-prepared	○	○	○	○	○
3) Calibration session was conducted effectively	○	○	○	○	○

Feedback on Administration

To what extent,	Excellent	Good	Neutral	Poor	Very Poor
4) Is the staff helpful, informed, and courteous?	○	○	○	○	○
5) Are the candidates treated respectfully and politely?	○	○	○	○	○
6) Is the exam adequately staffed?	○	○	○	○	○

Feedback on Venue & Facilities

To what extent,	Excellent	Good	Neutral	Poor	Very Poor
7) Are the facilities (e.g., seating, catering, secure storage) for the exam appropriate?	○	○	○	○	○
8) Is the soundproofing at each station sufficient?	○	○	○	○	○
9) Is the route between stations clearly marked?	○	○	○	○	○

10) Please provide comments on areas of strengths and constructive feedback for areas needing improvement

____
