# Supplementary material for: Stakeholder perspectives on the Emirati board comprehensive clinical examination: a mixed-methods multi-stakeholder evaluation
Source: Front Med (Lausanne). 2026 Oct 14;13:1900166. doi: 10.3389/fmed.2026.1900166 (PMC13713724; doi:10.3389/fmed.2026.1900166)
Supplement: Supplementary file 5 [file Datasheet5.pdf]

Instrument	Domain	Items (n)	Cronbach's α	McDonald's ω	Corrected item–total correlation (range)
Examinee questionnaire	Overall perception	7	0.891	0.897	0.576–0.826
Examiner questionnaire	Overall perception	13	0.943	0.947	0.544–0.812
QA General	Overall quality	7	0.881	0.906	0.581–0.788
QA Station	Educational Value	3	0.916	0.921	0.816–0.859
QA Station	SP Performance	4	0.934	0.940	0.819–0.883
QA Station	Examiner Performance	3	0.832	0.847	0.667–0.742
