# Supplementary material for: Stakeholder perspectives on the Emirati board comprehensive clinical examination: a mixed-methods multi-stakeholder evaluation
Source: Front Med (Lausanne). 2026 Oct 14;13:1900166. doi: 10.3389/fmed.2026.1900166 (PMC13713724; doi:10.3389/fmed.2026.1900166)
Supplement: Supplementary file 6 [file Datasheet6.pdf]

UAEU جامعة الإمارات العربية المتحدة United Arab Emirates University

Social Sciences Ethics Committee - Research / Course

Ethical Approval Letter

Date: 24/03/2026

This is to certify that application No:ERSC_2025_6404, titled:Transforming Clinical Assessment: A Retrospective Analysis of Stakeholder Feedback on the Emirati Board Comprehensive Clinical Exam for Continuous Improvement and Quality Assurance, submitted by Mohammed Al Houqani has been reviewed and approved by UAEU Social Sciences Ethics Committee - Research / Course on 17/06/2025.

***Sincerely*,**

Chair of the UAEU Social Sciences Ethics Committee - Research / Course
Research Ethics Sub-Committee
