# Supplementary material for: Stakeholder perspectives on the Emirati board comprehensive clinical examination: a mixed-methods multi-stakeholder evaluation
Source: Front Med (Lausanne). 2026 Oct 14;13:1900166. doi: 10.3389/fmed.2026.1900166 (PMC13713724; doi:10.3389/fmed.2026.1900166)
Supplement: Supplementary file 7 [file Datasheet7.pdf]

Supplementary Table S6. Examinee Feedback Form (n = 390) (n = 368)

Questionnaire Item	N	Mean ± SD	Positive Agreement (%)
Examiner interaction	368	3.93 ± 0.99	**72.3**
Station information	368	3.79 ± 1.17	**68.2**
Pre-CCE orientation	368	3.86 ± 1.22	**69.8**
Clinical judgment	367	3.99 ± 0.95	**75.2**
Clinical skills	368	3.89 ± 1.04	**73.1**
Clarity of instructions	368	3.84 ± 1.06	**69.6**
Overall organization & logistics	368	3.85 ± 1.19	**69.8**

Supplementary Table S7. Examiner Feedback Form (n = 390)

Questionnaire Item	N	Mean ± SD	Positive Agreement (%)
Information on CCE design	390	4.58 ± 0.66	**96.7**
Pre-CCE orientation	390	4.62 ± 0.66	**96.4**
Clinical skills	388	4.38 ± 0.79	**89.9**
Clinical judgment	390	4.43 ± 0.72	**90.8**
Electronic scoring	390	4.57 ± 0.68	**94.6**
Instructions	390	4.59 ± 0.67	**96.2**
Organization & flow	390	4.69 ± 0.59	**97.7**
Examiner support	390	4.73 ± 0.57	**97.9**
Soundproofing	390	4.75 ± 0.57	**97.7**
Candidate comfort	388	4.68 ± 0.61	**97.2**
Time allocation	390	4.54 ± 0.78	**91.3**
Assessment criteria	390	4.38 ± 0.84	**88.7**
Examiner autonomy	387	4.61 ± 0.66	**95.9**

Supplementary Table S8. QA General Feedback Form (n = 70)

QA General Feedback Item	N	Mean ± SD	Positive Agreement (%) (Good/Excellent)
Orientation and instructions provided to candidates and examiners were effective	70	4.77 ± 0.49	97.1
All necessary material before the start of the examination was well prepared	70	4.77 ± 0.49	97.1
Staff were helpful, informed, and courteous	70	4.91 ± 0.28	100.0
Candidates were treated respectfully and politely	70	4.91 ± 0.28	100.0
Examination was adequately staffed	70	4.86 ± 0.35	100.0
Examination facilities were appropriate	70	4.84 ± 0.37	100.0
Soundproofing at examination stations was sufficient	70	4.76 ± 0.49	97.1
Route between stations was clearly marked	69	4.74 ± 0.44	100.0

Supplementary Table S9. QA Station Observation Forms

**QA Station Observation Item**	**N**	**Mean ± SD**	**Positive Agreement (%) (Good/Excellent)**
Educational Value – Coverage of knowledge and skills	618	4.78 ± 0.55	97.6
Educational Value – Curriculum sampling	617	4.74 ± 0.53	97.7
Educational Value – Higher-order thinking	618	4.68 ± 0.64	96.4
Time allocation adequate	618	4.64 ± 0.68	92.2
Simulated Patient – Adequate guidance	406	4.77 ± 0.44	99.8
Simulated Patient – Patient comfort	387	4.79 ± 0.43	99.7
Simulated Patient – Patient well trained	396	4.76 ± 0.48	98.0
Simulated Patient – Standardized performance	383	4.72 ± 0.57	95.0
Examiner Performance – Adherence to script	615	4.80 ± 0.44	98.5
Examiner Performance – Courtesy and professionalism	615	4.86 ± 0.36	99.5
Examiner Performance – Standardization and consistency	554	4.77 ± 0.51	96.2
