# Supplementary material for: Stakeholder perspectives on the Emirati board comprehensive clinical examination: a mixed-methods multi-stakeholder evaluation
Source: Front Med (Lausanne). 2026 Oct 14;13:1900166. doi: 10.3389/fmed.2026.1900166 (PMC13713724; doi:10.3389/fmed.2026.1900166)
Supplement: Supplementary file 8 [file Datasheet8.pdf]

Table 1: Joint Display of Integrated Quantitative and Qualitative Findings Across Major Examination Domains

Domain	Quantitative Findings	Qualitative Themes	Integration Interpretation
**Orientation & Information**	Examinees reported lower mean scores (3.86), compared to examiners (4.60) and QA (4.77)	Unclear instructions, inconsistent expectations, gaps in pre-exam orientation	Lower examinee ratings are explained by variability in clarity and consistency of instructions, suggesting implementation differences across stations
**Logistics & Environment**	Examinees reported moderate scores (3.85), while examiners and QA reported high satisfaction (4.6–4.8)	Prolonged waiting times, fatigue, environmental inconsistencies (e.g., noise, flow issues)	Divergence reflects differences between system-level organization (highly rated) and lived examinee experience (affected by waiting and fatigue)
**Educational Value**	High ratings across all stakeholders (examinees 3.89; examiners 4.41; QA 4.71)	Clinically relevant stations, real-world applicability, appropriate assessment of higher-order skills	Strong convergence indicates shared perception of educational value and alignment with intended competencies
**Task Clarity**	Lower ratings among examinees mean 3.84 (linked to orientation domain)	Ambiguous instructions, mismatch between written and verbal expectations	Qualitative data explain quantitative variability, indicating that unclear task expectations contribute to inconsistent experiences
**Time Allocation**	Generally high ratings (QA 4.71), though slightly lower relative to other QA domains	Insufficient time in some stations, cognitive overload, prolonged pre-exam waiting	Overall satisfaction with timing was qualified by station-specific constraints affecting examinee performance.
**Examiner Behaviour**	High ratings across all groups (examinees 3.93; QA 4.81)	Professionalism widely reported; variability in prompting style and tone	General agreement on professionalism, with qualitative nuances highlighting inconsistencies in interaction styles
**Simulated Patient Performance**	High QA ratings (4.76)	Realistic portrayal, but variability in communication and consistency across examinees	High overall quality with some inconsistency, suggesting need for further standardization
**Assessment Fairness & Experience**	Lower overall perception among examinees	Lower overall perception among examinees	Lower overall perception among examinees

This joint display summarizes how quantitative and qualitative findings were integrated during interpretation. Quantitative and qualitative findings generally demonstrated convergence or complementarity across examination domains, with qualitative findings primarily providing contextual explanations for quantitative results.
