# Supplementary material for: Stakeholder perspectives on the Emirati board comprehensive clinical examination: a mixed-methods multi-stakeholder evaluation
Source: Front Med (Lausanne). 2026 Oct 14;13:1900166. doi: 10.3389/fmed.2026.1900166 (PMC13713724; doi:10.3389/fmed.2026.1900166)
Supplement: Supplementary file 9 [file Datasheet9.pdf]

AI-Assisted Organization of Qualitative Data: Workflow and Example Prompts

Purpose is to solely to assist with the preliminary organization of anonymized qualitative comments obtained from stakeholder questionnaires and structured post-examination debriefing discussions. AI was used only as an organizational support tool to facilitate management of a large qualitative dataset. It was not used to generate codes, identify themes, interpret findings, perform statistical analyses, or draw study conclusions. All analytical decisions remained the responsibility of the research team.

AI-assisted workflow

The workflow used during qualitative analysis is summarized below.

Step	Activity	Responsible Team
1	Export anonymized qualitative comments from REDCap and structured debriefing field notes.	Research team
2	Remove any personal or potentially identifying information before AI processing.	Research team
3	Submit anonymized comments to ChatGPT using structured organizational prompts.	Research team
4	Request grouping of similar comments according to operational topics without interpretation or theme generation.	ChatGPT
5	Review AI-generated groupings against the original comments and revise where necessary.	Research team
6	Independently code the original qualitative data.	Research team
7	Compare codes, resolve differences through discussion, and develop themes by consensus.	Research team
8	Integrate qualitative themes with quantitative findings during interpretation.	Research team
9	Final interpretation and reporting by the research team.	Research team

Example AI prompts

The following prompts illustrate how AI was used during the organizational phase of qualitative analysis.

Prompt 1: Organize the following anonymized stakeholder comments into groups based on similar operational issues or discussion topics. Do not interpret the comments, generate themes, or draw conclusions. Preserve the original wording wherever possible.

Prompt 2: Review these anonymized stakeholder comments and cluster similar responses relating to topics such as examination organization, logistics, task clarity, examiner interactions, simulated patient performance, time allocation, and overall examination experience. Do not perform qualitative analysis or interpret the findings.

Prompt 3: Present the grouped comments in a table showing the operational topic together with the corresponding original anonymized comments. Do not modify the wording of participant comments.

Prompt 4: Identify duplicate or highly similar comments to facilitate review by the research team without removing any original responses.

Researcher verification: All AI-generated outputs were independently reviewed by the research team before qualitative coding commenced. Coding, theme development, interpretation, and integration with quantitative findings were performed manually by the investigators using an inductive thematic analysis approach. AI-generated organizational outputs were treated solely as administrative aids and were neither accepted automatically nor used as evidence during interpretation. Any discrepancies between AI-generated groupings and researcher judgments were resolved through discussion and consensus among the investigators.

Role of Artificial Intelligence: The use of AI was limited to:

- organizing large numbers of anonymized qualitative comments;
- grouping similar responses to facilitate manual review;
- identifying potentially duplicated or closely related comments.

AI was not used for:

- qualitative coding;
- theme generation;
- interpretation of findings;
- statistical analysis;
- writing study conclusions;
- determining the final thematic framework.

All scientific interpretation and reporting remained the responsibility of the research team.
